# Livestock Network Analysis for Rhodesiense Human African Trypanosomiasis Control in Uganda

**DOI:** 10.3389/fvets.2021.611132

**Published:** 2021-06-28

**Authors:** Walter O. Okello, Christine A. Amongi, Dennis Muhanguzi, Ewan T. MacLeod, Charles Waiswa, Alexandra P. Shaw, Susan C. Welburn

**Affiliations:** ^1^Infection Medicine, Biomedical Sciences, Edinburgh Medical School, College of Medicine and Veterinary Medicine, University of Edinburgh, Edinburgh, United Kingdom; ^2^Commonwealth and Scientific Research Organization, Land & Water Business Unit, Canberra, ACT, Australia; ^3^Zhejiang University-University of Edinburgh Institute, Zhejiang University School of Medicine, Zhejiang University, Hangzhou, China; ^4^Biotechnical and Laboratory Sciences, Department of Biomolecular and Biolaboratory Sciences, School of Biosecurity, College of Veterinary Medicine Animal Resources and Biosecurity, Makerere University, Kampala, Uganda; ^5^The Coordinating Office for Control of Trypanosomiasis in Uganda (COCTU), Kampala, Uganda; ^6^Avia-GIS, Zoersel, Belgium

**Keywords:** HAT, cattle market, network analysis, livestock trade, risk, Uganda

## Abstract

**Background:** Infected cattle sourced from districts with established foci for *Trypanosoma brucei rhodesiense* human African trypanosomiasis (rHAT) migrating to previously unaffected districts, have resulted in a significant expansion of the disease in Uganda. This study explores livestock movement data to describe cattle trade network topology and assess the effects of disease control interventions on the transmission of rHAT infectiousness.

**Methods:** Network analysis was used to generate a cattle trade network with livestock data which was collected from cattle traders (*n* = 197) and validated using random graph methods. Additionally, the cattle trade network was combined with a susceptible, infected, recovered (*SIR*) compartmental model to simulate spread of rHAT (*R*_*o*_ 1.287), hence regarded as “slow” pathogen, and evaluate the effects of disease interventions.

**Results:** The cattle trade network exhibited a low clustering coefficient (0.5) with most cattle markets being weakly connected and a few being highly connected. Also, analysis of the cattle movement data revealed a core group comprising of cattle markets from both eastern (rHAT endemic) and northwest regions (rHAT unaffected area). Presence of a core group may result in rHAT spread to unaffected districts and occurrence of super spreader cattle market or markets in case of an outbreak. The key cattle markets that may be targeted for routine rHAT surveillance and control included Namutumba, Soroti, and Molo, all of which were in southeast Uganda. Using effective trypanosomiasis such as integrated cattle injection with trypanocides and spraying can sufficiently slow the spread of rHAT in the network.

**Conclusion:** Cattle trade network analysis indicated a pathway along which *T. b. rhodesiense* could spread northward from eastern Uganda. Targeted *T. b. rhodesiense* surveillance and control in eastern Uganda, through enhanced public–private partnerships, would serve to limit its spread.

## Introduction

Animal movements are integral to livestock trade but are not without risk for disease transmission. Infected Indian cattle in transit to Brazil reintroduced rinderpest to Europe in 1920, an infection that was eradicated worldwide in 2011 (1). The Office International des Epizooties (OIE) was established to mitigate risk and combat animal diseases (including zoonoses) at global level (1). The most infectious diseases for humans which are zoonotic in origin only serve to exacerbate risk for humans and animals (2), complicating trade and biosecurity within and between countries. Considerable efforts are put in place, underpinned by government policy to prevent disease spread, including attempts to develop a One Health approach to protect animal and human health (3). However, despite best efforts, these may be insufficient as evidenced by migration of Africa Rift Valley fever to Madagascar (4) and the struggle faced by Uganda over two decades to halt migration of *T. b. rhodesiense* HAT (rHAT) (5). Public–private partnerships were used to prevent impeding epidemic and spread of rHAT in eastern Uganda (6).

Since 2001, movements of infected animals from districts for which rHAT is endemic to new unaffected districts have spread rHAT around the shores of Lake Kyoga, toward the *T. b. gambiense* HAT (gHAT) focus in the north of the country (7–10). In 2008, 40% of cattle involved in inter-district trade were estimated to have been transported from rHAT endemic zones in the southeast to north and central districts (11).

Close examination of livestock movements and market networks offers the opportunity for understanding risk and exploring potential pathogen transmission. Trade is complex and dynamic and can be interrogated using complex network analysis (12–14); can accommodate bidirectional relations such as animal movement, trade, and contacts (15); and provides a theoretical framework for analysis of network properties and comparisons (16–18).

Contact network analysis has been used for modeling disease spread and to predict epidemics (19–21). Social network analysis (SNA) has been used to establish sexual contact relationships for human immunodeficiency virus/acquired immunodeficiency syndrome (22, 23) and has proved useful for studies of infectious disease transmission in livestock and wildlife. Studies include determining spread of tuberculosis in cattle (24) and in brushtail possums (25); *Escherichia coli* O157 in cattle (26); avian influenza in poultry (27, 28); and Foot and Mouth Disease in the UK (29–37). Livestock trade networks have been previously explored using SNA (38–40), particularly in Africa and in studies linking livestock trade to risk of zoonotic disease spread (41, 42).

This study explores cattle trade dynamics in eastern and northern Uganda regions to (1) understand cattle trade network topology and (2) evaluate the effects of disease control interventions on the spread of rHAT with varying infectiousness. Specifically, the study aimed to determine the role of the inter- and intra-district cattle trade in the potential spread of rHAT and identify key cattle markets for targeted disease surveillance and control.

## Method

### Study Site

This study was conducted in SE Uganda in Tororo and Namutumba districts. Vegetation cover in the area is mainly composed of savannah grassland interspersed with *Lantana camara* shrubs (43–45). The study area receives 1,200–1,500 mm of rainfall annually, which is bimodal in distribution. There are two wet seasons (March–May and September–November) and two dry seasons (December–February and June–August) (43). The daily mean minimum temperature is 15.8°C, and the mean maximum is 27.8°C (44). Agricultural economic activity comprises smallholder mixed farming, with over 80% of the population deriving their livelihood from agriculture (43) producing several different food and cash crops and integrating crop production with livestock keeping revised (46). The main reason for keeping cattle is as draft for crop cultivation; work oxen represent 36.5–43.7% of the cattle population (47, 48). Movement of untreated cattle is common in SE Uganda (49). A spatial study showed that predicted spread of endemic vector-borne and parasitic bovine infectious diseases common in these districts includes animal African trypanosomiasis (AAT), theileriosis (East Coast fever), babesiosis, anaplasmosis, heartwater, gastroenteritis, and fascioliasis (50, 51).

Tororo and Namutumba districts have been endemic for rHAT since the late 1980s (52) with human infective parasites identified in indigenous cattle in Tororo district since 1987 (53–58). *T b. rhodesiense* HAT has spread around the shores of Lake Kyoga causing significant human outbreaks associated with movement of infected animals (7, 9, 59) driven by a policy of restocking to assist districts further north, impoverished by war and generations of cattle raiding by the Karamajong in the 1980s and 1990s (11). Cattle raiding by the Karamajong depleted the livestock population in some areas to 3% of their original size (60), although not all districts in eastern and northern Uganda; these regions have similar agro-ecological zones, i.e., semiarid with subsistence farming of cattle, cassava, and millet (61). In Uganda, livestock traders move between districts and are not based within a specific district, thus providing a useful proxy for understanding cattle movement in most regions of Uganda.

### Sampling and Data Collection

**Figure 1:**
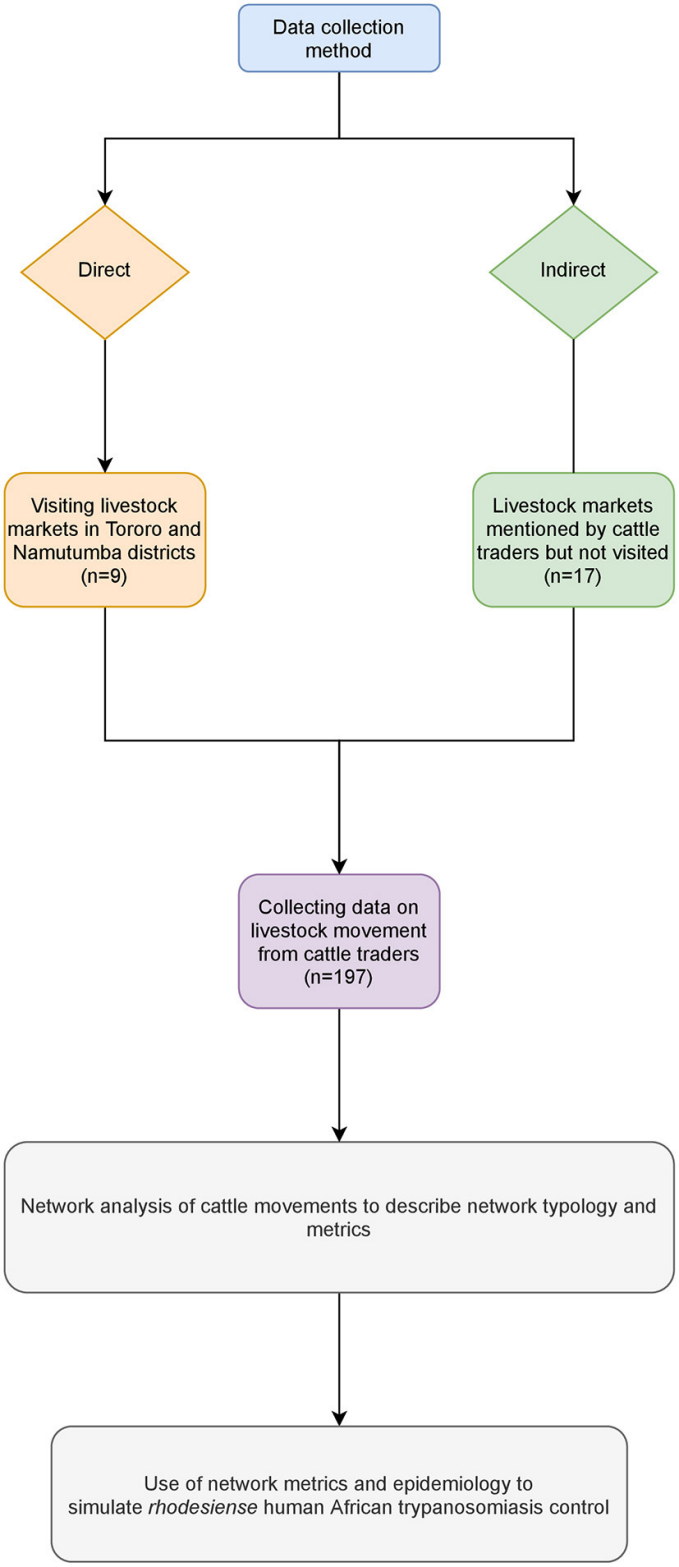
Cattle movement data collection and analysis methods.

**Figure 2:**
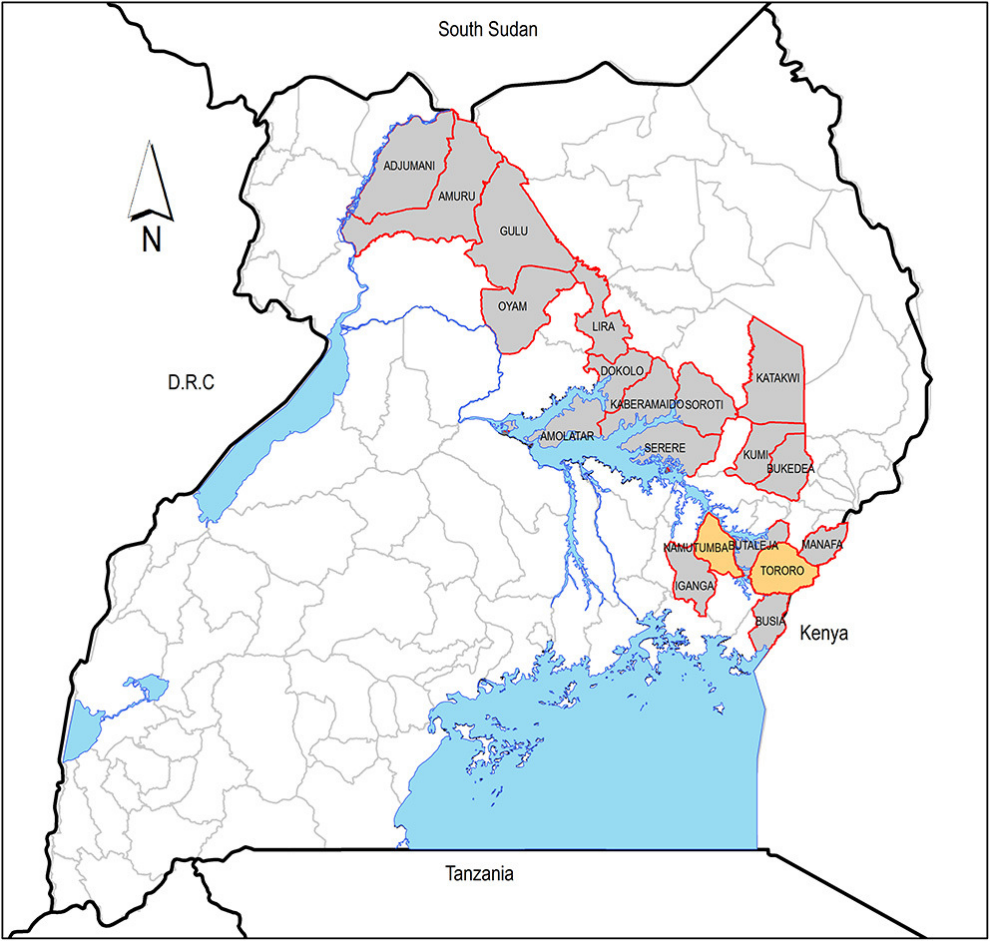
Districts in Uganda where cattle are mostly traded. The yellow districts (Namutumba and Tororo) are where data was collected from. Water bodies are shown in blue.

Authorized governmental livestock trade (small and large scale) takes place at defined market locations. These are local within districts and operate periodically under the jurisdiction of the local district livestock movement inspectors. All live livestock markets included in this study were identified from the records available at the district veterinary office. Livestock markets were visited on their respective market days and market transaction reports collected. Market transaction reports contained names of the trader and number of animals sold, but information on origin and destination was inconsistent. Therefore, information on animal movement was sought from cattle traders. Data was collected both directly (visiting the livestock markets in Tororo and Namutumba districts) and indirectly (livestock markets from other districts which were not visited but mentioned by the cattle traders). Figure 1 shows the flow of data collection. The livestock markets where data was directly collected are shown in Figure 2.

Cattle traders, through verbal consent, were interviewed using semi-structured interviews. Questionnaires were designed to capture interviewee information, livestock markets where cattle traders mostly sourced their cattle within the entire livestock trade cycle (annual), the livestock markets that these animals were sold into, and livestock market size. In total, all 197 traders present during the visit were cross-sectionally interviewed in all (*n* = 9) livestock markets in Tororo and Namutumba districts, SE Uganda. The origin and destination of the cattle as collected from this study have been provided (see Supplementary Material). Apart from collecting network data, we collected information on livestock market, cattle prices, and cattle trade dynamics using direct observation and conducted key informant interviews with local council authorities, cattle traders, and animal health providers.

### Data Analysis

SNA methods of (62, 63) were applied. Cattle markets represented the nodes (or actors), and the link (or tie) was represented by the connection of cattle markets through movement of cattle. Market attributes were determined by (i) size where big markets (assigned a value of 2) were represented by >20 cattle traders with >100 cattle traded weekly; small markets (assigned a value of 1) which were represented by >20 cattle traders with <100 cattle traded weekly; and (ii) past studies (secondary data) on *T. b. rhodesiense* prevalence in livestock (11, 64, 65). Data on HAT prevalence in cattle in Uganda was obtained from searching PubMed, EBSCO, and parasitology journal databases. The obtained secondary data for *T. b. rhodesiense* prevalence was fitted *via* beta and uniform distribution and Monte Carlo simulation to obtain 95% uncertainty interval (UI) in R (package = fitdistr) (66). The total value of actor (i.e., cattle market) attribute was weighted by assigning them sizes of the cattle market and prevalence of *T. b rhodesiense* obtained from the past studies to represent strength of a cattle market (node).

The cattle trade network in Uganda was evaluated by (1) describing the network typology and (2) identifying key cattle markets (key nodes) that potentially play a major role in disease spread and hence can be a major focus for disease control based on node centrality measures. Network typology was described using inter (network level metrics) and intra (node level metrics) network metrics and community detection. Inter-network metrics analyzed included the size of the network (total number of cattle markets and contacts that make up the network), density (i.e., measuring the degree of the contacts between pairs of cattle markets in the network), clustering coefficient (i.e., measuring the average probability of individual cattle markets being directly connected to one another in the network, hence measuring the tendency of the network to cluster), and modularity (measures strength of division of a network into communities, hence used for detecting community structure in a network) using the Clauset–Newman–Moore algorithm (62, 67, 68).

Intra-network metrics analyzed included cattle market connectivity (identifying the strong component of the network), centrality (degree of centrality, degree of betweenness and degree of closeness), and cohesiveness (i.e., identifying groups of cattle markets as part of a common structure of contacts such as k-core) (69, 70). The k-core describes the maximal subgroup in which each cattle market has at least degree k. The k is a metric for determining the coreness and therefore helps identify tightly interlinked groups within a network. Community detection was done using hierarchical clustering and community membership matrices including block modeling and structural equivalence (16, 71–73) to identify communities and overlap between them. Intra- and inter-network metrics were analyzed in R (package = igraph, package = sna) (74, 75) statistical computing version 3.2.2 (76). Density was computed using the formula in (77). Table 1 provides a summary of the network metrics including their epidemiological significance.

**Table 1:** Description of network and node level metrics.

**Metric**	**Description**	**Epidemiological importance**
**NETWORK-LEVEL METRICS**		
Size	Number of cattle markets (nodes) that make up the network. It enables comparison of the Uganda cattle market with other markets' random graphs.	Larger networks may have more subgroups that act as disease transmission bottlenecks within the group
Density	Degree of contact between pairs of cattle markets in the network	Disease transmission may occur faster in high-density networks
Eigenvector centralization	Measures the level of influence of a cattle market (node) within a network after assigning each a score.	Disease transmission occurs rapidly in networks with high eigenvector centralization
Modularity	Involves partitioning of the cattle network into well-connected subgroups	Disease transmission is slowed downed by the presence of subgroups
Clustering coefficient	Is the ratio of the number of edges (i.e., links) that occur between a cattle market's (i.e., node's) immediate neighbors and the maximum number of edges that could exist between them	High clustering may increase the frequency of disease spread
**NODE-LEVEL METRICS**		
Degree centrality	The number of edges (links) a cattle market (node) has.	Indicates whether a cattle market can be a source of infection (high out-degree centrality) or receive most of the infection from other cattle markets (high in-degree centrality)
Degree betweenness	Measures the extent to which a cattle market (node) lies on the paths between other cattle markets	Measures how frequently a given cattle market (node) can act as a bridge between other cattle markets (nodes) in the network. The higher the degree betweenness, the higher the potential of a cattle market to transmit the infection from a source cattle market
K-core	The k-core of a graph is the maximal subgraph made of nodes with degree k or more.	Can identify superspreaders or groups within a network which are vulnerable to a disease
**KEY ACTORS**		
Articulation point	Is a cattle market (node) whose removal disconnects the network	Can be targeted for disease control to enhance the resilience of the network

Cattle movement was set as directed (i.e., each cattle market has a direction associated with it) and weighted (i.e., using attributes to assign the importance of the links between cattle markets) since data obtained from livestock traders indicated the flow of cattle. Sensitivity analysis was included by setting cattle movement as undirected, i.e., cattle moving to a certain market and coming back to the original market. The study further analyzed clusters (communities) using links rather than nodes (78) within R (package = linkcomm) (79) statistical computing version 3.2.2 (76). By clustering links between the cattle markets, overlapping, and nested network structures, key cattle markets that form links across several clusters could be identified (80, 81).

### Validation and Simulation of Disease Outbreak and Control

Before conducting disease spread simulation on the network, the network data was first validated using (1) Erdös-Rényi random graphs with binomial distribution and (2) small-world networks *via* random rewiring (16). Specifically, this involved using observed nodes to generate a random Erdös–Rényi graph and that the observed network exhibited properties of small-world effects, i.e., creation of short paths between arbitrary nodes (16). Network validation using random graphs and rewiring recommended in instances where information on the entire network is not available (82).

Using the network typology, the spread of animal disease (using rHAT as an example) was simulated in the network to assess the effects of disease control interventions on disease transmission with varying infectiousness and related probability of transmission (β). This was achieved by using a stochastic susceptible, infected, recovered (*SIR*) compartmental model. The basic reproductive number (*R*_*o*_) for rHAT was obtained from previous studies (83, 84); average *R*_*o*_ of 1.287 was used in this study. Given that rHAT *R*_*o*_ was <1.5, it was used to represent a “slow” pathogen transmission. However, we also simulated a “fast” (*R*_*o*_ 3) disease transmission to compare with a “slow” one in the network. Probability of transmitting rHAT along the network (β) was calculated by dividing rHAT *R*_*o*_ with its infectious period in livestock which is on average 60 days (2 months) (84). The probability of transmission used in this study was 0.02 (1.287 divided by 60) and 30-time steps. In previous studies, it has been reported that combination of trypanocide treatment and insecticide spraying is effective, reducing rHAT transmission to *R*_*o*_ 0.0075 (85). Therefore, we reduced *β* to 0.000125 to simulate rHAT control using effective methods such as trypanocide treatment and insecticide spraying within the network. Assuming the same infectiousness period as rHAT (i.e., 60 days), we simulated disease control of a “fast” pathogen by reducing infectiousness by 50% (i.e., *R*_*o*_ 1.5 hence *β* 0.05), and a further 25% (i.e., *R*_*o*_ 0.75 hence *β* 0.0125).

## Results

### Characteristics of Livestock Markets and Cattle Trade

Trade at the major markets is the first tier of the livestock trade chain; subsequent tiers of trading buy livestock from fellow livestock traders to sell on as live animals, for slaughter, for breeding, or for supply of animal traction. At the first tier, livestock are sold and exchanged between different livestock markets within and outside the district of the market but most often in the same region. Subsequent tiers of trade are within the district where the first-tier livestock market is found. Most livestock traders interviewed traded in livestock reported sourcing animals from within their home or adjacent districts and including districts in the Busoga/Lake Victoria crescent rHAT focus such as Iganga and Busia (Figure 2). The cattle markets where cattle traders traded most of their cattle were both in eastern and northern regions of Uganda. The districts in eastern Uganda where cattle were mostly traded are shown in Figure 2, and these included Tororo, Namutumba, Soroti, Serere, Iganga, Busia, Manafa, Butaleja, Bukedea, Kumi, Katakwi, and Kaberamaido. The northern districts (see Figure 2) included Dokolo, Amolatar, Lira, Oyam, Gulu, Amuru, and Adjumani. The mean selling price according to cattle type was as follows: calves, United States dollar (USD) 37.8 (36.1–39.5), untrained young male for plowing, USD 90.3 (87.4–92.3), trained young male for plowing, USD 224.2 (182.7–267.2), cow, USD 207.7 (181.6–232.5), and adult male, USD 381 (275.8–495.2).

### Network and Node Metrics

**Figure 3:**
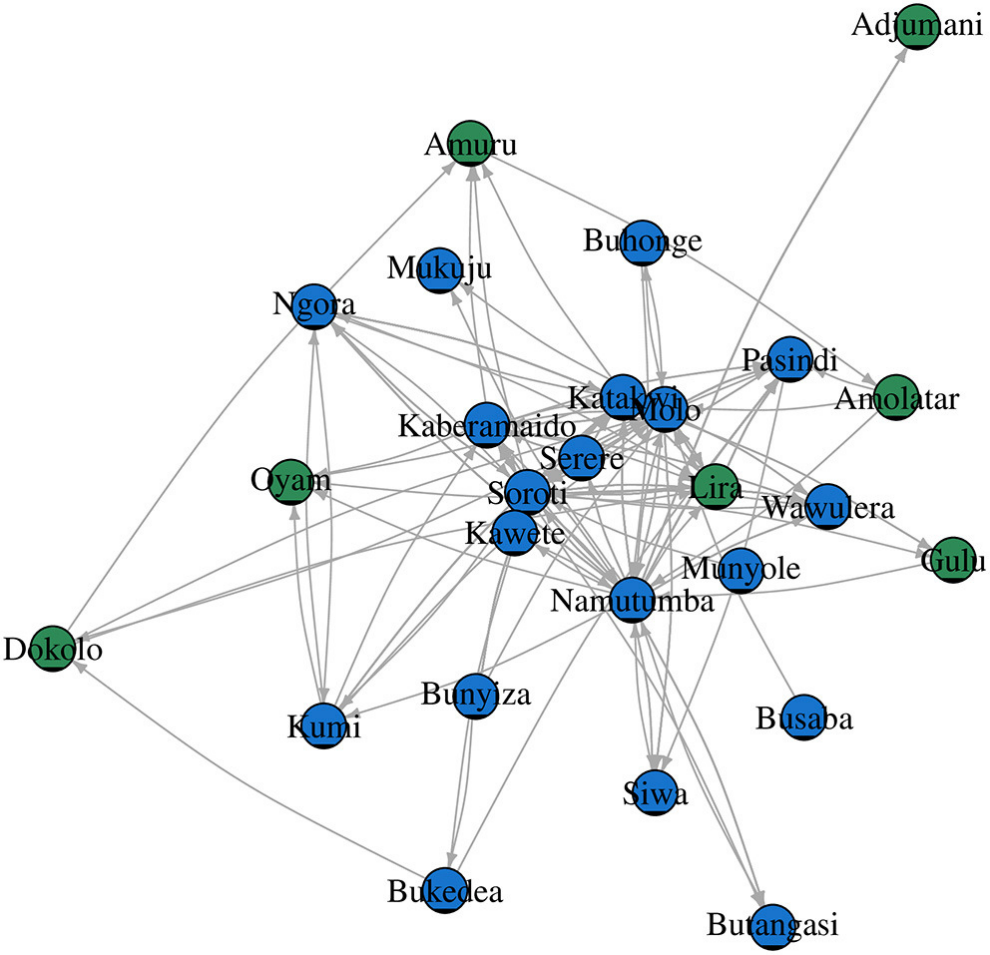
Cattle trade network in northern (green nodes) and eastern (blue nodes) Uganda.

**Figure 4:**
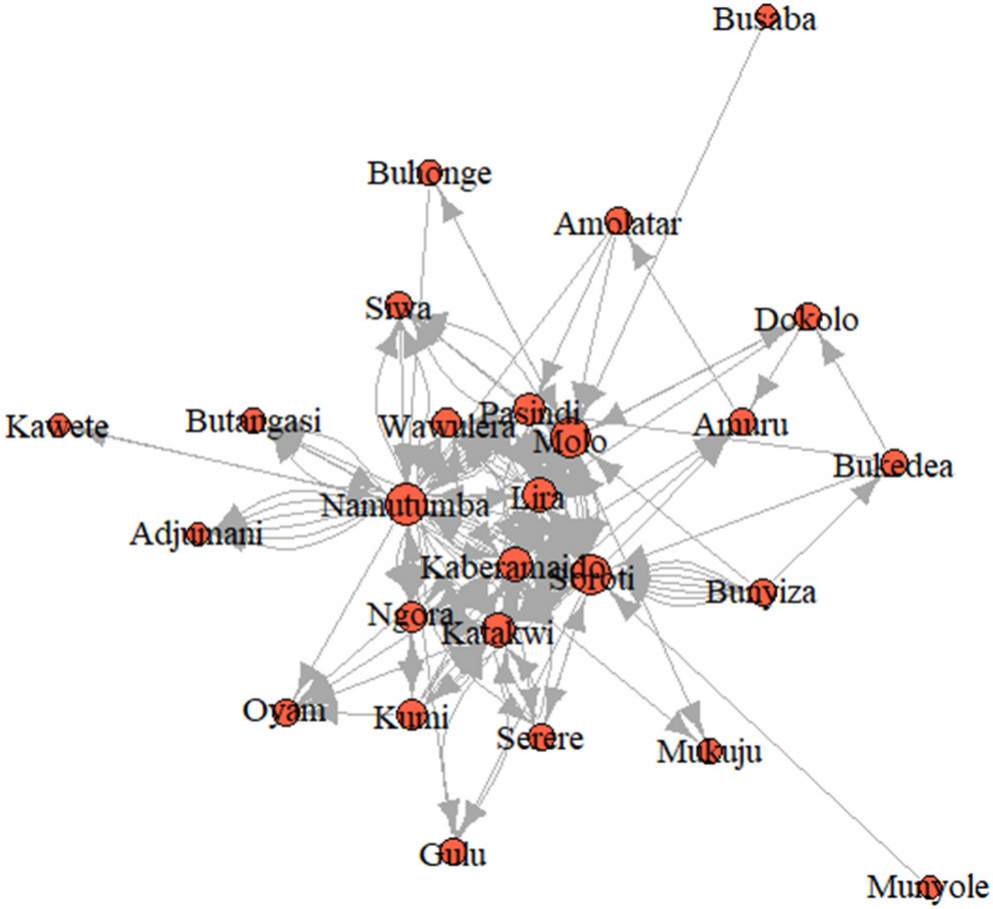
Weighted distance paths of the cattle trade network.

For SNA analysis, 197 traders were cross-sectionally interviewed in all (*n* = 9) livestock markets in Tororo and Namutumba districts, southeast Uganda. The cattle trade network (Figure 3) comprised of 26 cattle markets in both eastern and northern Uganda, 325 dyads (links between two cattle markets) and 197 links (Table 2) for which there were 60 mutual and 137 duplicated links. In addition, there was only one single connected component within the network. Weighted distances were also calculated to examine the length of all the shortest paths from or to the cattle markets in the network. The distance-weighted paths for the cattle markets are shown in Figure 4.

**Table 2:** Cattle trade network metrics in southeast and northwest Uganda.

**Metric**	**Values**	**Minimum**	**Maximum**
Number of cattle markets (nodes)	26.0	-	-
Number of links between cattle markets	197.0	-	-
Number of links between two cattle markets (dyads)	325.0	-	-
Density	0.0	-	-
Clustering coefficient	0.5	1.0	0.0
Average degree centrality	5.9	19.0	1.0
Average betweenness centrality	10.8	100.0	0.0
Average closeness centrality	0.0	-	-
Eigenvector centralization	0.3	-	-

Grouping the cattle markets using clusters and the Clauset–Newman–Moore algorithm, network modularity was 0.1. No isolated cattle markets existed in the network. Most cattle markets were weakly connected with a few being highly connected. Overall elementary graphical indices showed the density of the graph to be 0.006; dyadic reciprocity to be 1.7; edgewise reciprocity to be 1.6; and eigenvector of centralization to be 0.3.

The degree centrality score for each cattle market is shown in Table 3. Soroti livestock market in SE Uganda was shown to have the highest number of links and have a centrality score of 55.0, indicating the highest movement of cattle in and out of the district, followed by adjacent livestock markets in Namutumba (54.0) and Molo (51.0). Katakwi, Lira, Pasindi, and Kaberamaido showed a moderate flow of cattle in and out of the district. Ngora, Wawulera, Kumi, Bunyiza, Serere, Siwa, Adjumani, Mukuju, Buhonge, Buhangasi, Dokolo, and Amuru livestock markets had a relatively low movement of cattle in and out of the district.

**Table 3:** Cattle trade node metrics for all markets.

**Cattle market ID**	**Cattle market**	**Degree centrality**	**Betweenness centrality**	**Closeness centrality**	**K-cores**
1	Adjumani	6	0	0	6
2	Amolatar	4	20	0	4
3	Amuru	5	21	0	4
4	Buhonge	3	0	0	3
5	Bukedea	4	0	0	4
6	Bunyiza	9	0	0	8
7	Busaba	1	0	0	1
8	Butangasi	5	0	0	5
9	Dokolo	5	0	0	4
10	Gulu	8	2	0	8
11	Kaberamaido	27	16	0	19
12	Katakwi	33	29	0	17
13	Kawete	2	0	0	2
14	Kumi	11	0	0	10
15	Lira	32	9	0	19
16	Molo	51	118	0	19
17	Mukuju	2	0	0	2
18	Munyole	1	0	0	1
19	Namutumba	54	153	0	19
20	Ngora	17	0	0	15
21	Oyam	5	0	0	5
22	Pasindi	30	3	0	19
23	Serere	8	0	0	8
24	Siwa	7	1	0	7
25	Soroti	55	134	0	19
26	Wawulera	13	0	0	13

The degree of betweenness and closeness and the k-cores are summarized in Table 3. Namutumba had the highest degree betweenness followed by Molo and Soroti, respectively. Namutumba was also observed to have the highest degree of closeness followed by Soroti and Molo. The correlation between closeness and betweenness was 0.8. Animal diseases such as rHAT are most likely to pass through Namutumba district, i.e., diseases are most likely to come into Namutumba district and easily passed to other districts *via* the cattle trade network.

**Figure 5:**
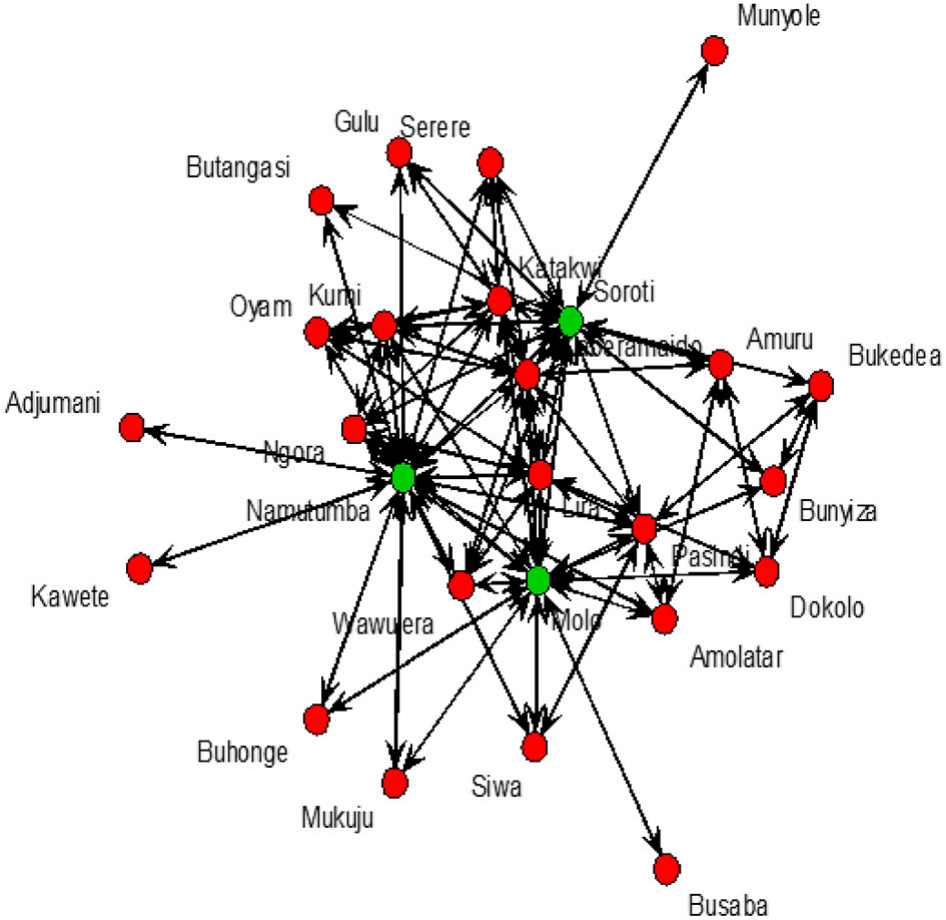
Cattle markets where *T. b. rhodesiense* is likely to be spread from. Soroti, Namutumba, and Molo shown by green dots.

Cattle markets with the highest k-cores were Kaberamaido, Lira, Molo, Namutumba, Pasindi, and Soroti. The analysis revealed several nesting cores. By limiting the number of k-cores, the members of the five-core, as a nesting core, were Soroti, Molo, Katakwi, Kaberamaido, Kumi, Lira, and Oyam. The five-core members may potentially be super spreaders of rHAT and are vulnerable to disease incursion. The key cattle market whose removal would disintegrate the network (articulation points) were Soroti, Namutumba, and Molo (Figure 5), representing key nodes where routine disease surveillance and control may be targeted to prevent spread of rHAT.

**Figure 6:**
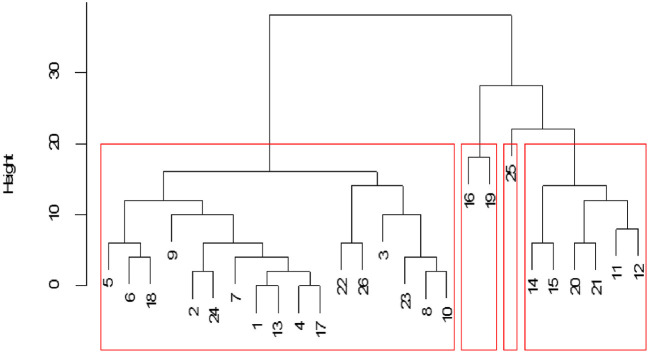
Cattle market structural equivalence within the network. Numbers represent cattle market identification (ID), and red boxes indicate the cluster.

**Figure 7:**
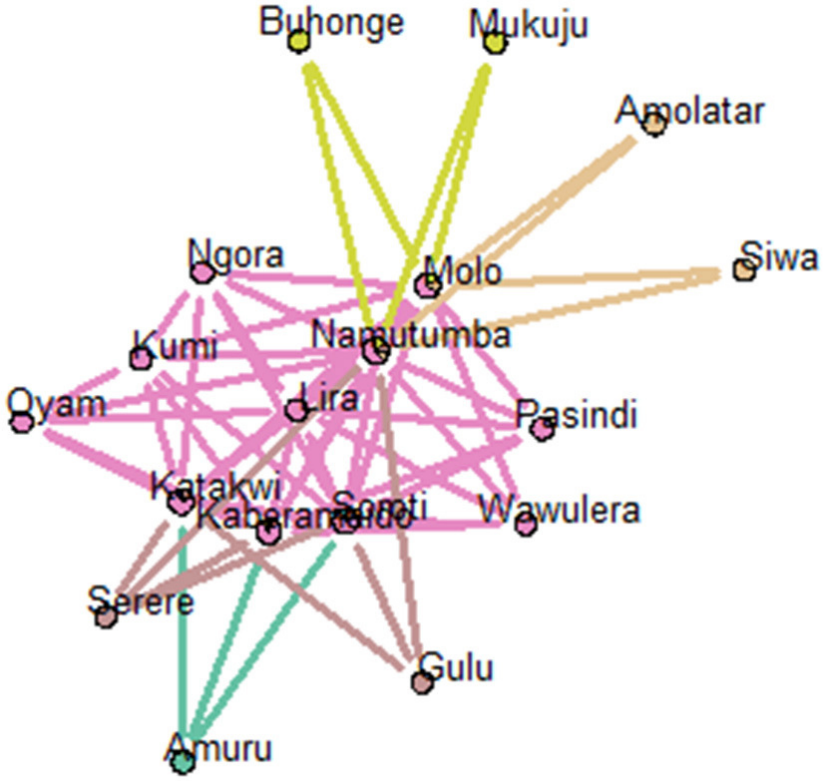
Visualization of link communities within the cattle network (using node pies). The fraction of the total number of edges that a node has in each community is depicted using a pie chart.

Examination of structural equivalence as shown in Figure 6 revealed that there were four clusters, indicating similarities in the structure of cattle trade for each cluster. As shown in Figure 6, cluster one was comprised of Bukedea (ID 5), Bunyiza (ID 6), Munyole (ID 18), Dokolo (ID 9), Amolatar (ID 2), Siwa (ID 24), Busaba (ID 7), Adjumani (ID 1), Kawete (ID 13), Buhonge (ID 4), Mukuju (ID 17), Pasindi (22), Wawulera (ID 26), Amuru (ID 3), Serere (ID 23), Butangasi (ID 8), and Gulu (ID 10). Cluster two was comprised of Molo (ID 16) and Namutumba (ID 19). Cluster three was comprised of Soroti (ID 25). Cluster four was comprised of Kumi (ID 14), Lira (ID 15), Ngora (ID 20), Oyam (ID 21), Kaberamaido (ID 11), and Katakwi (ID 12). Network block modeling, a measure of similarity using nodes, revealed no single block that connected all others. Extraction of link clusters *via* single hierarchical clustering, as measure of similarity using links, showed five clusters in the network with a maximum partition density of 0.42, the largest having 11 nodes. Additionally, there were five link communities in the cattle network as shown in Figure 7.

**Figure 8:**
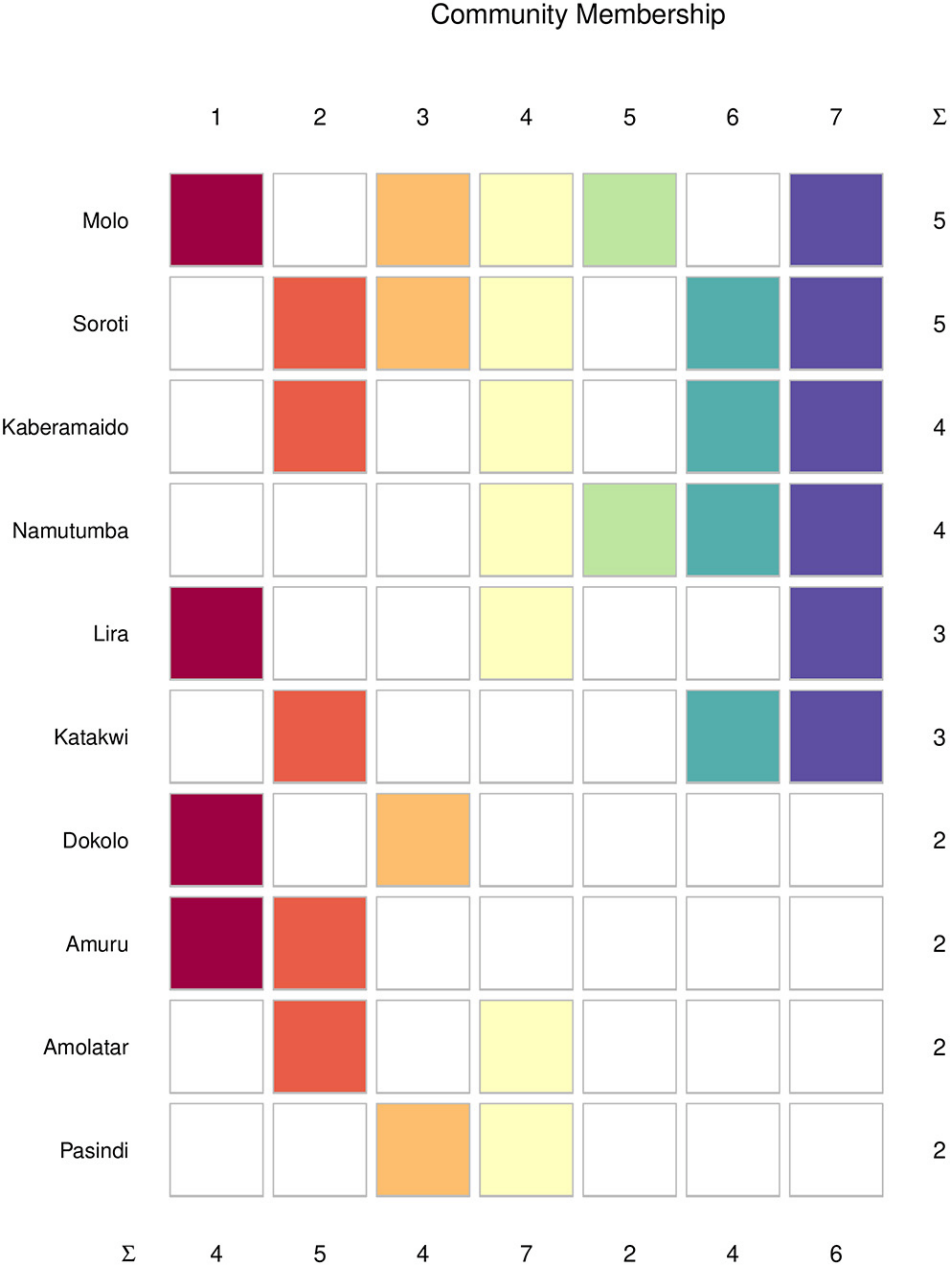
Community membership of the top (most) connected cattle markets in southeast and northwest. Colors indicate the community-specific membership, and the N-Ary summation (∑) shows the total number of cattle markets in each community.

From the community membership matrix, the most connected cattle markets (connected to five or more communities) were in the following order of connectedness: Molo > Soroti > Kaberamaido, Namutumba>Katakwi > Dokolo >Amuru >Amolatar > Pasindi (Figure 8). Livestock markets in SE Uganda comprised 66% of the top connected nodes in the cattle trade network. Limiting actor community membership for the top connected cattle markets to those belonging to three or more communities revealed Molo, Soroti, Kaberamaido, Namutumba, and Katakwi to be the most connected markets.

### Sensitivity Analysis

Sensitivity analysis comparing unweighted and undirected and weighted and directed cattle trade network showed some differences in the k-cores and the top connected livestock markets. K-cores for each actor were twice than for those of a directed network. The top connected nodes in the undirected network were Namutumba, Soroti, Kaberamaido, Katakwi, Molo, Amuru, Lira, Pasindi, Kumi, and Ngora. Therefore, in the undirected cattle network, SE Uganda livestock comprised of 78% of the top connected nodes. The articulation points (cut points), which were Namutumba, Molo, and Soroti, were the same in both directed and undirected networks.

### Simulated Disease Transmission

**Figure 9:**
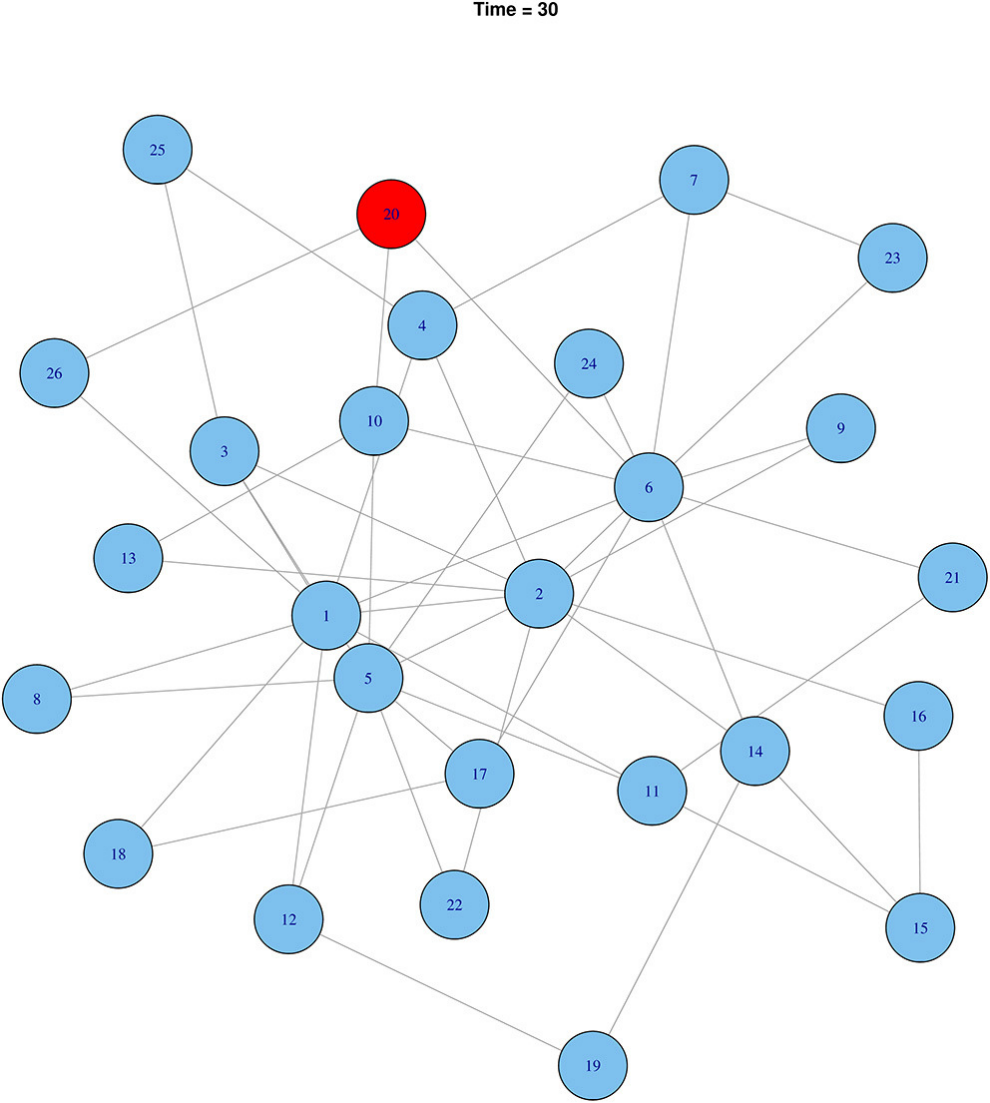
Effect of controlling rHAT when using effective control methods after 30-time steps. Infected nodes are shown in red and uninfected in blue.

**Figure 10:**
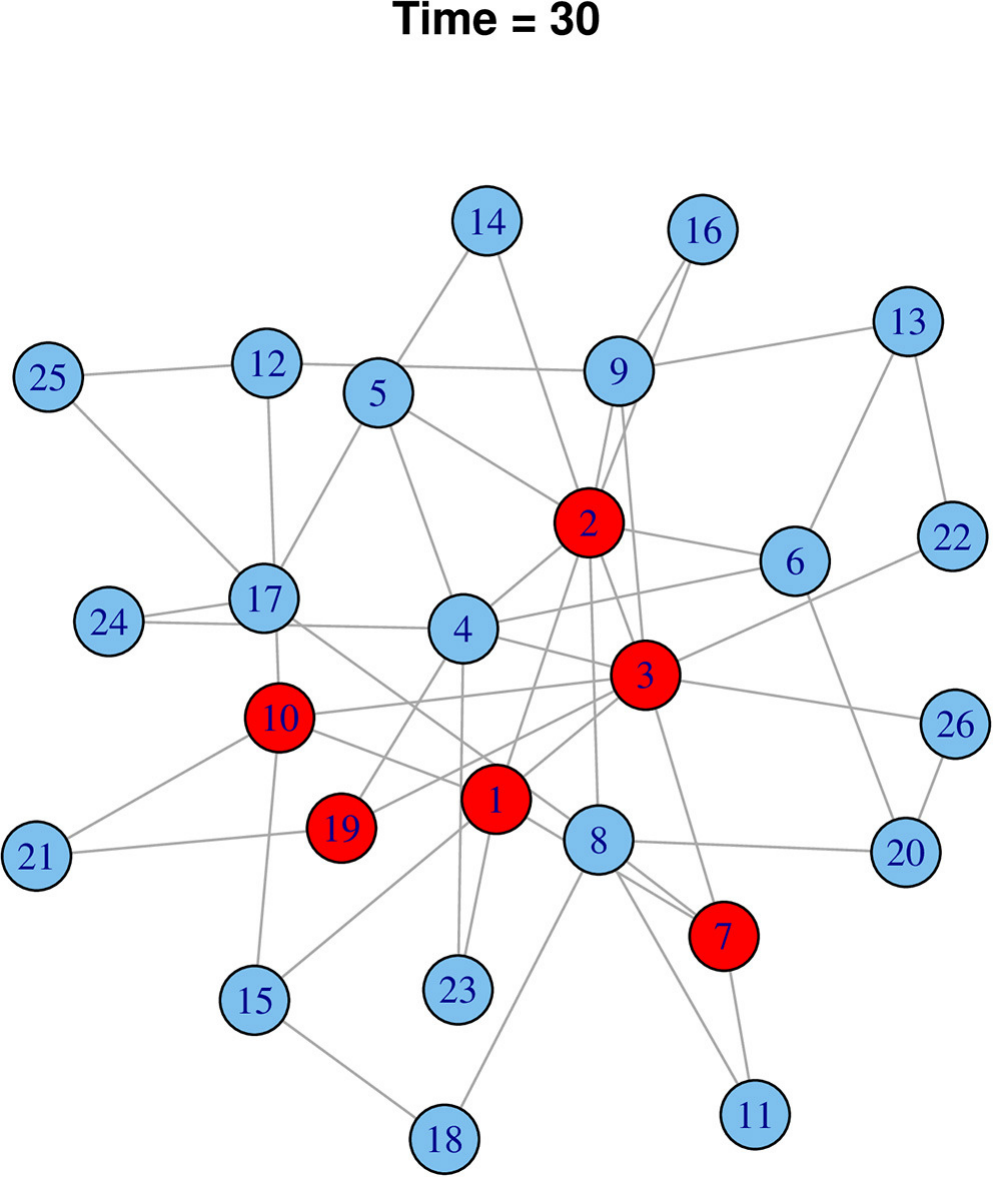
Effect of controlling “fast” pathogen (*R*_*o*_ 3) when infectiousness is reduced to 25% (*R*_*o*_ 0.75) after 30-time steps. Infected nodes are shown in red and uninfected in blue.

Starting from a random cattle market, it was simulated that rHAT would have spread to six cattle markets at the 30-time step. Using effective rHAT control methods such as combined cattle treatment and spraying would reduce the transmission to only one cattle market (Figure 9). In comparison to a highly infectious pathogen, 20 cattle markets would have been infected at the initial 30-time step (i.e., *R*_*o*_ 0.05) reducing to 12 cattle market when infectiousness was reduced by 50% (i.e., *R*_*o*_ 0.025), and eventually six cattle markets when infectiousness was reduced by a further 25% (*R*_*o*_ 0.0125) (Figure 10).

## Discussion

*Trypanosoma brucei rhodesiense* has rapidly spread through the cattle trade network in Uganda, moving infection progressively northward. Previous work confirmed the contribution of livestock movements through formal livestock markets and restocking in disease spread. Uganda is a source of meat for the East African community, Democratic Republic of the Congo, and Southern Sudan (11).

Human infective parasites were first observed in indigenous cattle in Tororo district in 1987 (52). *T. b. rhodesiense* HAT rapidly spread around the shores of Lake Kyoga causing significant human outbreaks that were associated with movement of infected animals (7, 9, 58) driven by a policy of restocking districts impoverished by war and generations of cattle raiding by the Karamajong. Restoration of peace in northwest Uganda and South Sudan is a significant driver for the trade and sale of livestock for meat between the two countries (11). Another potential driver of cattle is cattle prices. In this study, we found that cattle prices are influenced by biophysical characteristics and demand for animal traction, with adult male cattle and young male trained for plowing fetching the highest prices. Further analysis of factors underlying livestock movement is still required to be done.

Analysis of livestock movement data has been shown to be valuable mostly in high-income countries where such data are routinely collected. In developing countries, data on livestock movement detailing origin, destination, number of cattle sold, cattle prices, etc., are limited and in most cases unavailable. Equally, resources are not always available to routinely collect and collate such data for decision-making. By collecting cattle movement data from cattle traders, this study shows that it is possible to use expert domain knowledge to construct a network. The value of conducting livestock network analysis in resource-limited settings lies in the possibility of identifying key cattle markets that can be targeted for routine disease control, reducing costs and disease impact. Additionally, simulating animal disease spread enhances understanding of the effectiveness of disease control methods. For example, in this study, we show that for “slow” pathogens like rHAT, effective treatment strategies can sufficiently reduce the spread of rHAT. It has been shown that treatment of cattle using diminazene aceturate and spraying for tsetse flies to protect cattle against trypanosomiasis is effective and has high net benefits (86–88). Compared to “slow” pathogens such as rHAT, control of “fast” pathogens within the network may be problematic and costly requiring a wider coverage of cattle markets or a highly effective control method or methods. This is because even when disease infectiousness and transmission is reduced to 25%, the number of infected cattle markets was still substantial.

The cattle network examined here can be categorized as both connected and heterogeneous. Heterogeneity coupled with a low clustering coefficient, asymmetry. and high skewness as found in this study is typical of scale-free networks (89). The low density (0.3%) and clustering coefficient (0.5) indicate that the cattle trade network has a random pattern, making it difficult to predict a future likely source of rHAT. Cattle and poultry trade network studies in Madagascar (4, 41) showed a similar weakly connected network with low density and clustering coefficient. The average centrality value for the cattle trade network in this study was low, indicating that cattle are being moved through few connections, most likely as a result of majority livestock traders in Uganda operating at small scale. While low connection within the trade network raises the probability of low disease detection, it does offer opportunity to control disease spread within the network.

Examination of degree centrality and betweenness revealed that Soroti had a high cattle movement in and out the district, whereas most cattle passed through Namutumba. Therefore, rHAT and other infectious diseases can easily start at this district or be passed to other districts. Equally, most animal diseases can easily be transferred to Namutumba district and passed to other districts. In the past, Soroti was an epicenter of rHAT outbreak in 1999/2000 which was linked to Brooks Corner livestock market (currently in Serere district) (7).

The study also identified a core group (five-core) of cattle markets that are vulnerable to rHAT and perhaps other animal diseases and may act as superspreaders. The members of the core group were the most connected markets, with the highest flow (in and out) of cattle, and they were found in the eastern and northwest districts of Uganda, increasing the probability of spread of rHAT from the endemic southeast parts to the non-endemic northern parts of Uganda. Members of the core group in eastern Uganda included Namutumba, Molo, Soroti, Pasindi, Wawulera, Ngora, Kumi, and Katakwi whereas northern Uganda markets included Oyam and Lira. This core group would maintain infection and serve as an epicenter for the spread of infection to other cattle markets in Uganda, particularly if the original infection was from Namutumba, Soroti, or Molo livestock markets.

Cattle markets that connect southeast and northwest Uganda play a key role in the spread of pathogens. Consequently, Government policy dictates that cattle sold at markets should be treated with trypanocidal drugs prior to sale to prevent movement of *T. b. rhodesiense*-infected cattle. Implementation of this policy, however, is not straightforward. It is the responsibility of the purchaser to pay for both the trypanocides (~US$0.30 per animal for treatment with curative diminazene aceturate to US$0.75 each animal for treatment with isometamidium chloride which has a 3-months prophylactic effect) along with the veterinary fees for administering the treatment (~US$0.30 per animal). Most cattle markets are not perimeter secure, resulting in buyers frequently avoiding extra costs by leaving. Another challenge is that animals purchased for subsequent slaughter should not be treated with trypanocides or should only be slaughtered after the withdrawal period of such trypanocides. Aside from the requirement to treat cattle in livestock markets, Uganda law also decrees that any animal destined to move across district boundaries has the correct permit for passage between the specified districts. Ideally, permits should be issued by qualified veterinary personnel subject to animals passing an inspection (examination of clinical manifestations of any communicable disease). Permit records should be kept by the District Veterinary Officer's office from the market of issue, with duplicates dispatched to the Ministry of Agriculture Animal Industry and Fisheries, Entebbe. However, implementation of all the required laws is challenging. Therefore, from a practical point view, network analysis can be used to inform risk-based and targeted disease surveillance and control to circumvent some of the challenges in implementing disease control laws.

This study had limitations. First, livestock markets and cattle traders in northern and some parts of eastern Uganda were not interviewed, as the study was focused on rHAT and lacked resources to expand to other parts of the country. This resulted in a relatively smaller sample size which may affect the cattle network metrics. Second, the study relied on past *T. b. rhodesiense* prevalence as an attribute given that no blood samples were taken from cattle. Consequently, further research on livestock markets as well as sampling for *T. b. rhodesiense* is recommended. Third, the cattle network does not incorporate dynamic patterns such as seasonality, thereby limiting its complexity; longitudinal collection of cattle movement within a set period, e.g., 1 year, is required. Further limitations include use of a simple epidemiological model to simulate disease control; sophisticated modeling may make substantial differences in disease transmission more apparent.

This study recommends (i) control through chemotherapy and spraying of cattle with tsetse-effective insecticides and targeted surveillance of rHAT in key cattle markets (nodes) such as Namutumba, Soroti, and Molo cattle markets as opposed to untargeted disease control that may be costly, (ii) further targeted and routine surveillance in cattle markets in eastern and northwest Uganda to detect the presence of rHAT in cattle, and (iii) additional collection and analysis of livestock movement data from more cattle markets to understand animal disease risk. Spraying of cattle with deltamethrin using the restricted application protocol in addition to cattle treatment with curative trypanocides at the point of sale (e.g., in the cattle markets) is recommended by (7, 51). Trypanocidal drugs capable of temporarily clearing cattle of the human infective parasite are well-understood and widely available; tsetse-fly-targeted insecticides to prevent reinfection of cattle are also well-understood and widely available (89–97). The restricted application approach (RAP) to insecticide use at markets will reduce costs and is practically feasible (98). However, farmers require support for management of disease and policy to treat animals for the prevention of spread of diseases such as trypanosomiasis and tick-borne diseases needs to be reinforced (99, 100). Furthermore, indigenous cattle are predominantly kept under traditional communal grazing management and are either tethered or grazed on communal pastures. Under these management systems, cattle are exposed to continuous tsetse and tick challenge and the new strains of tsetse and tick-borne diseases (trypanosomiasis, anaplasmosis, babesiosis, and theileriosis) imported are difficult to contain following their introduction.

## Conclusion

*Trypanosoma brucei rhodesiense* can potentially be spread both within southeast and between this region and northwest Uganda by cattle trading. Targeted *T. b. rhodesiense* surveillance in cattle markets in southeast and northwest Uganda would enable early disease detection. Reinforcement of government policy for treatment of cattle at the point of sale through trypanocidal treatment and spraying to protect them from reinfection should be prioritized in eastern Uganda to limit spread of infection. The combined impact of these two interventions (i.e., trypanocidal injection and insecticide spraying), through public–private partnerships, will reduce the risk of reinfection caused by cattle moving into rHAT previously affected as well as unaffected regions of Uganda.

## Data Availability Statement

The original contributions presented in the study are included in the article/Supplementary Material, further inquiries can be directed to the corresponding author/s.

## Ethics Statement

The studies involving human participants were reviewed and approved by Makerere University College of Veterinary Medicine Animal Resources and Biosecurity ethical review board, and by the Uganda National Council for Science and Technology (approval number HS1336). The patients/participants provided their written informed consent to participate in this study.

## Author Contributions

WO was responsible for the conception, design, collection, drafting, and analysis of data. DM and CA were involved in the conception of the study and data collection. AS was involved in the conception, design, analysis, and drafting the manuscript. SW and CW were involved in the conception of the study, design, and revision of the manuscript. EM was involved in the design and drafting of the manuscript. All the authors read and approved the final version of the manuscript.

## Conflict of Interest

The authors declare that this study received funding from European Union's Seventh Framework Program. SW, CA, and CW declare that they received funding from UK aid. The funders were not involved in the study design, collection, analysis, interpretation of data, the writing of this article or the decision to submit it for publication. AS was employed by AVIA-GIS. The remaining authors declare that the research was conducted in the absence of any commercial or financial relationships that could be construed as a potential conflict of interest.
